# Dynamics of Pathogenic Fungi in Field Hedges: Vegetation Cover Is Differentially Impacted by Weather

**DOI:** 10.3390/microorganisms10020400

**Published:** 2022-02-09

**Authors:** Pauline Dentika, Harry Ozier-Lafontaine, Laurent Penet

**Affiliations:** INRAE, UR1321 ASTRO Agrosystèmes Tropicaux, 97170 Petit-Bourg, Guadeloupe, France; pauline.dentika@gmail.com (P.D.); harry.ozier-lafontaine@inrae.fr (H.O.-L.)

**Keywords:** *Colletotrichum gloeosporioides*, field hedges, *Psidium guajava*, disease dynamics, barrier effect, leaf age, drip tip, tree height, weather effects

## Abstract

Landscape effects might impede or increase spore dispersal and disease risk for crops, as trees and hedges buffer winds and can behave as spore traps, therefore limiting diffusion of fungi, or, on the contrary, behave as disease relay once vegetation is infected and become inoculum sources. In this study, we investigated weekly prevalence of the pathogenic fungus *Colletotrichum* *gloeosporioides* on guava tree leaves, differentiating impacts of leaf height on tree, age, and location within leaf. We first estimated differences in prevalence for each covariate, and then related infection rates to weather effects during the year. Our results highlighted a great variance of prevalence among individual trees, and a lower contamination of tree tops, as well as a tendency for greater odds of infection in tips of young leaves compared to older ones. Last, we show evidence that individual tree contaminations are associated with different disease dynamics: early and dispersal-based, late and growth-based, as well as with intermediate dynamic ranges. Pathogen infection dynamics will thus be greatly impacted by cover characteristics at local scale, and tree cover should not be perceived as homogeneously driving disease levels.

## 1. Introduction

Crop diseases are a major limitation of food production [1], and while previous decades relied heavily on chemical control of pathogens and antagonists [2], transition toward more sustainable food production will require designing new control practices and pathogen attenuation strategies. Classical panel of disease management is broad, from developing resistant varieties or multiline strategies with diverse resistance and resilience levels to disease [3], controlling environmental triggers of epidemics via agronomy (till or no till effect on soil microfauna and flora [4], and potential irrigation impact on microclimate and thus pathogens [5]; yet, more recently, landscape has emerged as a potential complementary tool in diseases and pests control [6,7,8]. Indeed, landscape effects have a wide spectrum [9], from indirect effects mediated by diversity of ecosystem services to more direct impacts relating to barriers to dispersal of antagonists [10] or proactive shelter and predation with demographic impacts [11]. Considering potential effect of antagonists on plants, from alteration of reproductive systems to productivity [12,13,14], landscapes may thus have transcending effects both locally and at greater scales, yet with complex impact [15].

Higher level effects of landscape pertain to diffusion of crop antagonists (barrier effect) [16], while local effects will have more demographic-based impacts, depending on whether they can significantly decrease or increase antagonist populations (host skill) [17]. Both consequences may also have complex relationships, as potential increase in demography may not necessarily increase disease or pest damage risk homogenously in the neighboring fields [18], especially since dispersal is usually strongly affected by barrier effects. Furthermore, these effects will depend on the nature of antagonists, as crop pests tend to have decreased attack patterns due to predation in edges with biodiverse wild strips or older hedges [19,20], while fungi may actually land on suitable hosts when trapped in hedges and strips, thus turning landscape into a local source of inoculums and potential disease relay [21]. Thus, for fungi, landscape may hinder diffusion of spores in the fields, but may prove only a short buffering lapse for more generalist pathogens with broad host range skill.

In this study, we focused on spore trapping effect (spores are retained in trees rather than landing on crops) and potential for local installation in hedges. We thus investigated the generalist pathogen *Colletotrichum gloeosporioides*, known for its broad host range [22,23,24,25,26,27] and ability to produce (anthracnose) disease in numerous crop species around the world, both in temperate and tropical environments, and locally on yams [28]. We assessed infection of *Colletotrichum gloeosporioides* weekly on a bush tree commonly growing in hedges or field strips in the Caribbean region, namely guava, across both “lent season” (drier season) and “rain season”. We recorded weather conditions in order to assess how climate is impacting leaf infection rates. Specifically, we aimed to address how local tree factors would interact in terms of fungus prevalence, and investigated the impact of leaf age (young vs. old) [29,30,31] and leaf location on tree (bottom, middle, and top), and since guava trees have drip tip leaves susceptible to top-down contamination by spore leaching during rains [32,33], we also assessed differences in prevalence in three leaf areas (near stem, middle leaf, and near drip tip). We therefore aimed to answer the following question: how do individual trees within hedges behave in terms of spore receipt/trapping of spore clouds in the air, and how would infection dynamics translate in terms of further risk for crops? We tried to delineate meteorological impact, specifically factors influencing dispersal (e.g., wind, rains) and those rising infection success of *Colletotrichum* (e.g., humidity, temperature).

## 2. Materials and Methods

We sampled leaves of guava on three individual neighboring fields distant by several hundred meters, every week, for 45 weeks. Our focus was more on weather effects over time; we therefore decided to study medium-term inoculation rates on a smaller sample (three individuals) over a longer period. Moreover, guava trees are often found in small populations of a few trees around field edges rather than large demographic groups, and our study thus reflects local circumstances more closely. Three leaves were sampled on each tree every time, beginning at bottom, then moving a third of circumference and sampling at middle height, then moving another third of circumference and sampling atop of the tree. Leaves were alternately picked as young and then old throughout weeks and throughout the season. Leaf age was differentiated as follows: old leaves are darker green, often demonstrating traces of damage (herbivory or dry necrosis spots) and are located deeper on ramets, while young leaves are light green and fresh/recent and without signs of damage, and they are located at proximal tips of ramets. Back in the lab, leaves were rinsed in short baths of diluted bleach solution, methanol, and decontaminated water, respectively [34]. Then, three leaf areas were cut, near the stem, mid-leaf, and near the drip tip. For each of these leaf areas, two pieces were cut for replication, and one piece was placed on a Petri dish with simple agar media and the replication was placed on a Petri dish with S medium [34] to favor growth of *Colletotrichum* over other fungi; these steps were processed in sterile conditions under a laminar flow cabinet (model LRF 48). Both replicates were nevertheless recorded as either positive (one or two *Colletotrichum* strains identified) or negative (no *Colletotrichum* growth in either piece). Petri dishes were placed for incubation time under 12 h light (under Osram T8 L 36 W/865 Lumilux DaylightG13 neons) at room temperature (22–28 °C) and assessed for presence of *Colletotrichum* under the microscope after five days. Prevalences were estimated as the ratio of *Colletotrichum* occurrence within sample divided by the total week sample.

In addition to calculating prevalence, we recorded the following weekly weather factors for the whole sampling period: average humectation duration (hereafter, DH, in decimal hours), precipitations or average daily rains and maximal rain (hereafter, RR and RRX respectively, in mm), minimal and maximal recorded humidity (hereafter, UM and UX respectively, in %), duration time of humidity levels >90% (U9, in decimal hours), minimum and maximum temperatures (respectively TN, TX, in °C), and weekly average wind speeds and maximal wind speeds (V and VX, respectively, in m.s^−1^). When compiling weather data matrix, factors were reported either as late (values for the sampling week) or early (values for the week preceding the sampling week, and noted with −2 suffix, e.g., for early rains, values for the week preceding the sampling are encoded RR2).

As a first approach, we ran a logistic regression model to explain prevalence as a function of the different categories: leaf age, leaf area, leaf location within tree, and trees, including interactions between leaf age and leaf area to account for the specific nature of leaf milieu. As a complement to our first approach (logistic regression), we performed a second exploratory, entirely different, two-step analysis as follows. In the first step, we used the “random forests” classification method to evaluate the importance of each of 20 weather variables (10 variables, each duplicated as an early and a late measurement) on 12 different sets of measurements, producing 12 random forest models: young leaf, old leaf, tip, mid-leaf, stem, tree bottom, middle tree, tree top, three models for individual trees (A, B, C), and a general model encompassing global prevalence estimate. This first step returned a 20 × 12 matrix of estimated “variable importance” (see Results section), which, in the second step, was itself subjected to principal component analysis in order to extract its main trends, with the 20 weather variables serving as data points and the 12 random forest models serving as PCA coordinates: we divided the weather variables into “early” versus “late” and into “growth” versus “dispersal” subsets in order to visualize the combined effects of each subset. Our aim was to describe meteorological factors in relation to the assessed models and aim for estimating weather factors effect on *Colletotrichum* prevalence in general. All analyses were produced using R software [35], including random forest package for the random forest analyses.

## 3. Results

**Figure 1:**
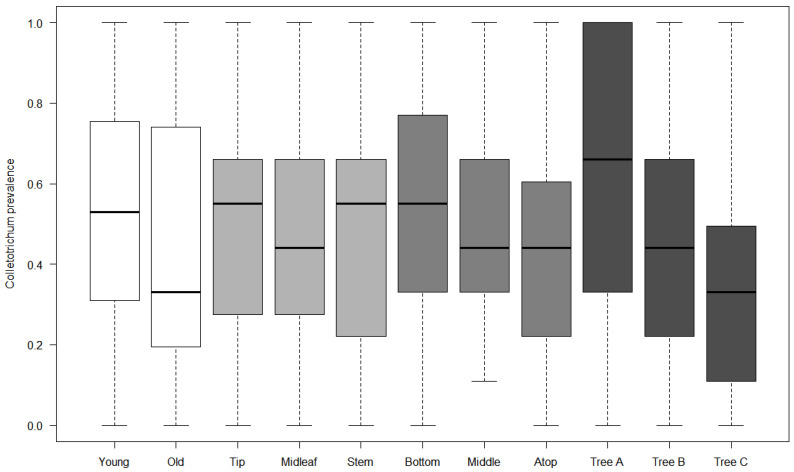
Global prevalence of *Colletotrichum gloeosporioides* for leaves and tree factor levels. Each level is illustrated with its own color: in white, leaf age (young vs. old leaves); in light grey: location on leaf (tip area, mid-leaf, near the stem); in grey: tree height (bottom, middle, and top of tree); in dark grey: individual tree variation (trees A, B, and C). Only trees and top of tree are significantly different within factor class, other factors are shown for pattern illustration purposes.

**Figure 2:**
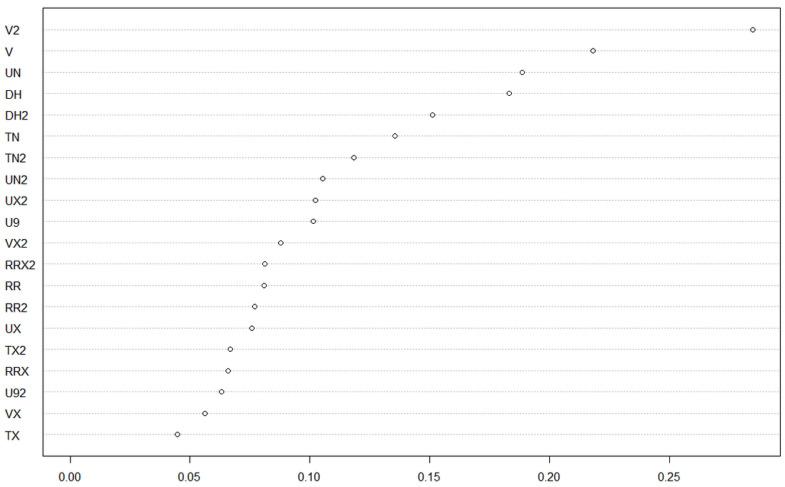
Rank analysis of covariate impact on prevalence in the random forest, example from fitting general prevalence of *Colletotrichum* on all sample trees (last column of Table 2). Meteorological covariates are ranked by average increase in node purity in tree models from the forest, i.e., its impact on variance at the node. Covariates with a 2 in their names are reflecting weather conditions from the week preceding the prevalence estimate, while those without are weather conditions from the same week as the prevalence estimate.

Our general logistic regression model highlighted mostly a great variation between individual trees regarding prevalence (Table 1), and almost no category had a prevalence significantly different from baseline categories, except for the “top of tree” category, which had a significantly smaller prevalence rate, while the interaction between leaf age and tip location was marginally significant, with a greater prevalence than expected by chance in tip area of young leaves. While the differences in prevalence were not significantly different, observed patterns of variation were grossly following a trend of contamination within trees by rain flow after spores were deposited on trees: tip and stem locations on leaves had greater rates of inoculation by *Colletotrichum*, and middle and top of trees had lower rates than the bottom of trees (Figure 1). Strikingly though, the tendency was a greater prevalence for young leaves than for old leaves overall. Our random forest models yielded poor results, indicating that meteorological conditions were insufficient proxies for contamination rate estimates, probably because the ultimate driver of epidemics is environmental spore cloud, and weather only contributed indirectly as conductive conditions for disease, and not as a direct causal factor. The best models explained only about 16–17% of variance (young leaf, bottom tree, and tree A), and the other models had a continuum of explained variance down to single digits, suggesting that only a couple of meteorological covariates were really impacting the dependent in the different models, while the others were unfortunately fitted over random noise in data. Since we were interested in ranking factors more than predictive ability of models, we kept the forest model assessment of covariate impact on prevalence (example, Figure 2; estimates of increase in node purity are given in Table 2 for every model).

**Figure 3:**
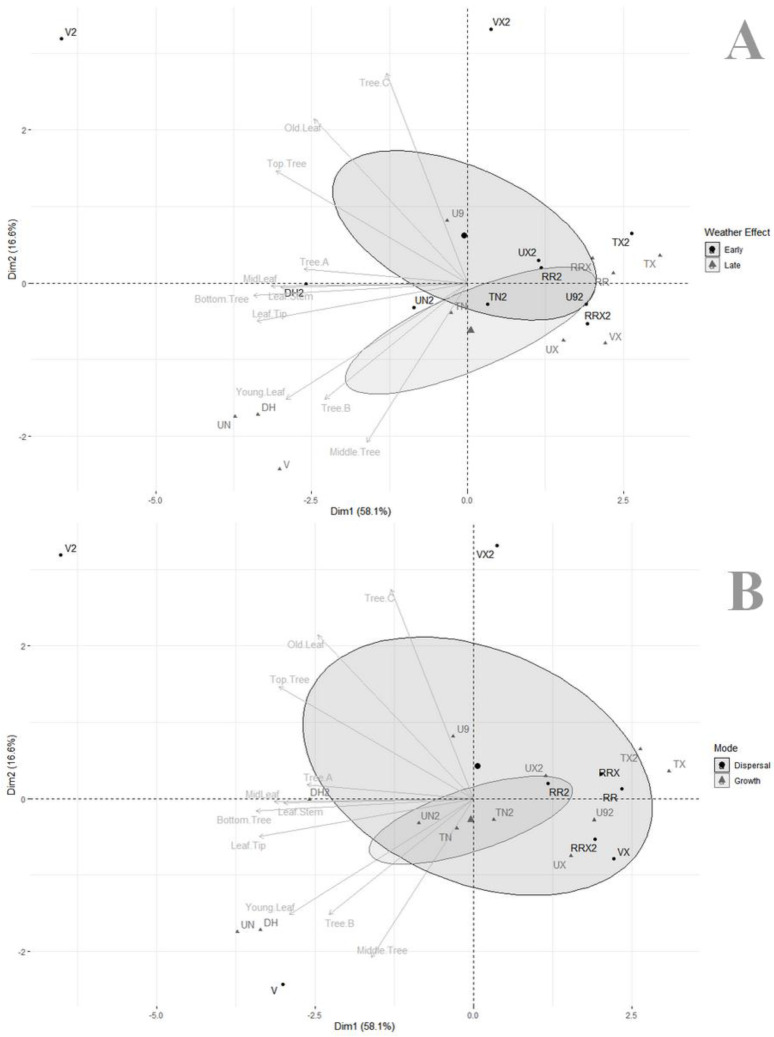
Principal component analyses of covariate impact in random forest models, illustrating impact of (**A**) weather effect (early vs. late) and (**B**) mode of impact (involved in dispersal vs. involved in fungal growth) (data from Table 2).

The principal component analysis based on variable importance from our random forest models (Table 2) was illustrated with either grouping meteorological factors as early vs. late (Figure 3A), i.e., pattern of weather effects from the previous week versus pattern of weather from the same week as the prevalence estimate, or grouping meteorological factors as more involved in dispersal (e.g., rain, wind) compared to those more involved in fungal growth (e.g., temperature, humectation) (Figure 3B). The two first axes of the PCA captured 74.7% of total variance. PCA divided covariates with an important rightwise pool of factors with very low correlations to individual model impacts (TX, TX2, RRX, RR, UX2, RR2, U92, RRX2, TN2, UX, VX), explaining the initial poor fit of our random forests (i.e., factors without actual impact on fungal prevalence). On the other hand, a few covariates were interspersed leftwise to their relative effects on specific models (V2, VX2, U9, DH2, UN2, UN, DH, V), and they each had diverse impact on models.

A first series of models were sharing important similarities (top tree, old leaf, tree C) and were more associated with V2 and VX2 and, thus, had a pattern correlation to early weather effects (Figure 3A) and dispersal (Figure 3B). In contrast, a second series of models (young leaf, middle tree, tree B) were more associated with UN2, UN, DH, and V and had a pattern of correlation to late weather effects (Figure 3A) and were more related to fungal growth (Figure 3B). The third series of models (all leaf location models, bottom tree, and tree A) were very closely related to each other and strongly associated with DH2, and also had an intermediate position between the first two series.

## 4. Discussion

We monitored *Colletotrichum* over 45 weeks on three guava trees, trying to assess its prevalence on old vs. young leaves, location on leaves (stem vs. mid-leaf vs. tip), and on trees (bottom, middle, and top). Estimated prevalence mostly did not differ among categories, except for a huge variation between individual trees, and top of trees being significantly less inoculated than the other parts of trees (Figure 1), and prevalence was marginally significant and greater for young leaf tips. Assessment of meteorological covariates demonstrated that trees presented different disease dynamics: a tree demonstrated important lag in *Colletotrichum* presence and followed a pattern closely related to old leaf and tree top model with early inoculation and importance of dispersal, another tree was more impacted by late inoculation and fungal growth factors more typical of young leaf and middle tree models, and a last tree exhibited intermediate characteristics, more typical of tree bottom dynamics (Figure 3). Our results thus suggest that factors involved in the different parts of trees and age and leaf characteristics seem to differ and contribute differentially to the global dynamics of pathogen instalment and further spread. We will review how these differences might interfere with a naive vision of homogeneity within cover and may make contribution to spore clouds and disease dynamics more complex.

Older leaves had smaller *Colletotrichum* prevalence than young leaves. This result is in contradiction with the view that younger leaves are generally better protected against antagonists, mostly against herbivores [36], as a consequence of possessing due chemicals at greater concentration [37,38]. This is indeed perceived in the literature as the tradeoff between protecting and producing new organs [39]. In the context of pathogen attacks, such chemical defenses in younger leaves may actually prove less efficient or highly dependent on fungus ability to manage chemically complex niches. A second argument might also be that young leaves generally have softer and more tender tissues and smaller cuticles, which make them possibly more vulnerable to fungal inoculation [40], and, while age was not a significant factor in differences in prevalence, assessment of weather factors yielded different models of infection relating to leaf age, with model of prevalence on young leaf being closely associated with covariates reflecting fungal growth, while model of prevalence on old leaf was more associated with factors involved in pathogen dispersal ability (Figure 3).

There was no impact of leaf area regarding prevalence, but the interaction between area and age revealed a marginally significant effect on prevalence, with young leaves being more inoculated than expected near the drip tip. Drip tips are indeed leaf adaptations allowing the plant organ to safely evacuate pathogenic spores away during rains (and in tropical environments, to remove epiphytic propagules in the same way [41]). Our results suggest that the function is indeed behaving correctly but as a consequence also increases odds of fungi to grow at this place, especially in young leaves. Overall, indeed, parts of the leaves with greater prevalence were near the stem or near the tip (Figure 1). While this might be a convenient means of spore disposal, it might also increase downflow contamination, as further suggested by both the prevalence pattern observed with tree height (increasing infection rates on average from tree top to bottom) and the close proximity of leaf area models to bottom tree model (Figure 3).

The different tree height categories were markedly different in terms of prevalence, and top of tree had significantly lower fungal prevalence (Table 1), while middle and bottom trees had more *Colletotrichum*. These results were grossly unsurprising, given the potential for downflow contamination following rains. On the other hand, they also revealed part of pathogen instalment dynamics, since the middle tree model was more associated with late winds, while the top of tree model was associated with early winds (Figure 3). This confirmed that trees are behaving as spore traps and are indeed depositing spores via long-distance wind dispersal. It also suggests a possible latent effect of wind for tree tops (early winds, i.e., winds preceding prevalence estimates by a week). In this case, contaminations of tree tops might happen indirectly via middle trees and might indicate that contaminations from rains (via splashes, see [42]) are occurring too, though at a slower rate than downflow contamination. It is important to take note that spore trapping by field hedge trees is probably slowing down crop contaminations by retaining spores that might otherwise have ended up inoculating crops, but once pathogens have been installed in these trees, field margins become at stake for further disease propagation.

Last, our results also highlighted huge variation in individual tree prevalence. Some trees had contamination models closer to certain component models (e.g., tree C model shared similarities to old leaf, and somewhat, to a lesser extent, to tree top models; while on the other hand, tree B was very close to young leaf and middle tree models, Figure 3). Leaf age and leaf area, and tree height as well, seem to have fairly different dynamics and are impacted differently by meteorological factors, possibly in relation to mechanistic conditions (e.g., tree height) or even organic characteristics (e.g., leaf age). All these factors combine a bit differently in each case, resulting in different pathogen dynamics too, and this may translate as differential contributions to spore clouds available. How this overall contributes to disease spread should be investigated more thoroughly, in order to assess whether disease patterns at landscape scales are actually impacted and how they might contribute to the dynamics of disease in crops.

## Figures and Tables

**Table 1:** Logistic regression model of *Colletotrichum* prevalence. Significant factors are highlighted in **bold**, marginally significant factors in *italics*. ** is for *p* < 0.01, *** is for *p* < 0.001.

	Estimate	Std. Error	Z Value	Pr (>|z|)
(Intercept)	0.9496	0.2058	4.614	3.95× 10^−06^ ***
**TreeB**	**−0.7568**	**0.1510**	**−5.012**	5.40 × 10^−07^ ***
**TreeC**	**−1.3174**	**0.1538**	**−8.568**	<2 × 10^−16^ ***
Leaf.AgeYoung	−0.2177	0.2138	−1.018	0.30853
Leaf.AreaStem	−0.1632	0.2161	−0.756	0.44995
Leaf.AreaTip	−0.1632	0.2161	−0.756	0.44995
Tree.HeightMiddle	−0.2242	0.1496	−1.499	0.13384
**Tree.HeightTop**	**−0.4550**	**0.1509**	**−3.015**	0.00257 **
Leaf.AgeYoung:Leaf.AreaStem	0.2277	0.2994	0.760	0.44703
*Leaf.AgeYoung:Leaf.AreaTip*	*0.5075*	*0.2999*	*1.692*	*0.09062*

**Table 2:** Variable importance for meteorological factors as extracted from the different random forest models, and their classification as early or late, and mode of impact on fungal life cycle. Values indicate average increase in node purity of the factor in the test model, i.e., its average impact in predicting fungal prevalence.

	Weather Effect	Mode	Young Leaf	Old Leaf	Leaf Tip	MidLeaf	Leaf Stem	Bottom Tree	Middle Tree	Top Tree	Tree A	Tree B	Tree C	Tree Global
DH	Late	Growth	0.306	0.168	0.240	0.146	0.328	0.288	0.255	0.197	0.373	0.209	0.126	0.183
DH2	Early	Growth	0.144	0.338	0.234	0.132	0.174	0.295	0.130	0.165	0.530	0.258	0.078	0.151
RR	Late	Dispersal	0.072	0.127	0.088	0.097	0.075	0.101	0.077	0.098	0.099	0.134	0.090	0.081
RR2	Early	Dispersal	0.087	0.187	0.187	0.090	0.061	0.116	0.137	0.116	0.170	0.111	0.126	0.077
RRX	Late	Dispersal	0.110	0.142	0.096	0.102	0.058	0.085	0.099	0.119	0.139	0.113	0.125	0.066
RRX2	Early	Dispersal	0.114	0.075	0.152	0.084	0.035	0.122	0.104	0.071	0.264	0.107	0.087	0.081
TN	Late	Growth	0.173	0.142	0.146	0.140	0.124	0.143	0.169	0.177	0.346	0.156	0.129	0.136
TN2	Early	Growth	0.125	0.127	0.186	0.102	0.083	0.108	0.222	0.147	0.297	0.118	0.197	0.118
TX	Late	Growth	0.051	0.096	0.085	0.058	0.057	0.051	0.077	0.090	0.113	0.101	0.118	0.045
TX2	Early	Growth	0.064	0.094	0.092	0.067	0.068	0.074	0.078	0.107	0.141	0.097	0.163	0.067
U9	Late	Growth	0.148	0.128	0.150	0.191	0.128	0.235	0.057	0.230	0.213	0.122	0.150	0.101
U92	Early	Growth	0.137	0.097	0.137	0.125	0.057	0.099	0.098	0.065	0.213	0.074	0.091	0.063
UN	Late	Growth	0.361	0.143	0.273	0.241	0.230	0.307	0.111	0.238	0.206	0.356	0.104	0.189
UN2	Early	Growth	0.185	0.152	0.181	0.175	0.167	0.188	0.141	0.196	0.175	0.191	0.143	0.105
UX	Late	Growth	0.090	0.103	0.114	0.077	0.106	0.167	0.084	0.088	0.137	0.231	0.072	0.075
UX2	Early	Growth	0.050	0.180	0.120	0.058	0.192	0.142	0.063	0.131	0.112	0.226	0.108	0.102
V	Late	Dispersal	0.420	0.138	0.241	0.228	0.109	0.344	0.260	0.146	0.219	0.236	0.155	0.218
V2	Early	Dispersal	0.254	0.428	0.295	0.270	0.295	0.391	0.112	0.438	0.546	0.184	0.363	0.285
VX	Late	Dispersal	0.103	0.090	0.117	0.052	0.078	0.109	0.131	0.087	0.084	0.163	0.087	0.056
VX2	Early	Dispersal	0.114	0.276	0.134	0.110	0.089	0.180	0.058	0.176	0.060	0.114	0.437	0.088

## Data Availability

Data are available on request from the corresponding author.
